# Airway complications after lung transplantation: Perioperative risk factors and clinical outcomes

**DOI:** 10.1016/j.jhlto.2025.100315

**Published:** 2025-06-06

**Authors:** Siddhartha G. Kapnadak, Kathleen J. Ramos, Rachel Flodin, Sanaa Mansoor, Kyle Bilodeau, Peter Beidler, Erika D. Lease, Ryan Thomas, Richard Dubois, Jay Pal, Michael S. Mulligan

**Affiliations:** a Division of Pulmonary, Critical Care, and Sleep Medicine, Department of Medicine, University of Washington, Seattle, Washington; b Division of Cardiothoracic Surgery, Department of Surgery, University of Washington, Seattle, Washington

**Keywords:** lung transplantation, airway complications, antifibrotics, immunosuppressants, intensive care unit

## Abstract

**Background:**

Airway complications after lung transplantation are common and contribute to worse outcomes. There are limited data documenting perioperative risk factors that could be mitigated to reduce risk. Our objectives were to (1) assess the impact of pretransplant disease–modifying medications and post-transplant hypotension, hypovolemia, and mechanical ventilation on the risk of airway complications; (2) evaluate the association of airway complications with post-transplant lung function and survival.

**Methods:**

One hundred and forty-five bilateral lung transplant recipients at our center were included. Demographics, pretransplant medications, post-transplant intensive care unit variables, and lung function were compared between recipients who did vs did not develop airway complications. Post-transplant survival was estimated using Kaplan-Meier analysis.

**Results:**

Forty-eight (33.1%) recipients (75% male) developed airway complications. There were no significant associations in pretransplant exposure to prednisone (including by dose), other immunosuppressants, or antifibrotics, alone or in any combination, with the development of airway complications. There were no differences in ventilation pressures, but recipients with airway complications had higher peak vasopressor-inotropic scores (18.0 vs 13.0, *p* = 0.021), lactate levels (9.1 vs 6.8, *p* = 0.017), need for hemodialysis (22.9% vs 10.3%, *p* = 0.042), and net fluid balance at 48 hours (10.6 vs 8.9 liters, *p* = 0.028), respectively, compared to those without. Airway complications were associated with significantly worse survival (HR 2.74 [95% CI 1.35, 5.55], *p* = 0.004) and lung function (peak forced expiratory value in 1 second 74.8% vs 86.3% predicted, respectively, *p* = 0.013).

**Conclusions:**

Postoperative hypotension and hypoperfusion are associated with increased risk for airway complications after lung transplantation. Airway complications are associated with poor outcomes, and further studies are needed to delineate risk-mitigation strategies.

## Background

Lung transplantation (LTx) can improve survival and quality of life for patients with advanced lung disease but outcomes remain suboptimal. Due to a tenuous vascular supply during and immediately following surgery, airway complications (ACs) remain one of the most common postoperative problems and represent a major source of morbidity. While the impact of ACs on lung function impairment is unclear and associations with survival have varied in single-center studies, 2 meta-analyses have demonstrated increased 1- and 5-year mortality.^1^, ^2^

Understanding risk factors for ACs after LTx is an important step toward improving outcomes, particularly if any can be modified to mitigate a recipient’s risk. Several studies have demonstrated risk factors inherent to a patient or their disease course, including male sex,^1^, ^2^ infectious colonization,^1^, ^3^, ^4^, ^5^ reduced telomere length,^6^ preoperative critical illness,^1^, ^2^ postoperative prolonged mechanical ventilation,^7^, ^8^ and acute rejection.^2^, ^9^ While these certainly promote knowledge regarding ACs, they are less helpful in terms of prevention strategies, and unfortunately, literature describing modifiable perioperative risk factors in the modern era is quite limited. For example, in the pretransplant setting, it is logical that immunosuppressants and antifibrotics could impact the risk of early airway infection and anastomotic healing,^10^ but granular data analyzing the composite risks of these medications by dose or in combination, as often used in practice, are lacking. In the post-transplant setting, although reduced blood flow to the anastomoses due to hypoperfusion or higher mechanical ventilation pressures could increase the risk of ACs,^11^, ^12^ there are minimal data describing these phenomena in the modern era, and it is unclear whether risk could be reduced with changes in fluid management or ventilation strategies.

Our goal was to investigate modifiable perioperative risk factors for ACs in a contemporary cohort. In the pretransplant setting, our focus was on lung-disease–modifying medications, including corticosteroids, other immunosuppressants, and antifibrotics. In the post-transplant setting, our focus was on insults that could contribute to anastomotic hypoperfusion, and we hypothesized that early postoperative hypotension and hypovolemia would increase the risk of ACs.

## Methods

All bilateral, first-time lung transplants at our center between January 2021 and December 2023 were included. Follow-up was available through December 2024. The University’s Institutional Review Board approved the study (STUDY00020064). Recipient data were retrieved from the electronic medical record, including demographic variables nearest to LTx and allocation scores at the time of transplant. Notably, the study period spanned transition from the United States’ lung allocation score (LAS) to the composite allocation score (CAS). Therefore, the match LAS was recorded for transplants before, and the last CAS medical urgency subscore for transplants after March 9, 2023. To allow allocation score comparisons between eras, patients were first ranked according to their LAS or CAS, creating 2 separate rank lists, then separated based on within-group rankings into LAS or CAS tertiles, then tertiles combined to create full-cohort tertiles, including patients from both eras (first tertile = highest LAS or CAS).

Medications were taken from the most recent list before LTx with attention to corticosteroids (including dose), other immunosuppressants, and antifibrotics. To assess composite risks, patients were classified (mutually exclusively) as being on: none, pretransplant prednisone alone, other immunosuppressants alone, antifibrotic alone, antifibrotic plus prednisone, antifibrotic plus other immunosuppressant, prednisone plus other immunosuppressant, or prednisone plus other immunosuppressant plus antifibrotic.

As a marker of postoperative hypotension, vasopressor-inotropic scores (VIS) were calculated as previously described^13^ upon arrival to the intensive care unit (ICU), and as a peak within the first 24 hours. Infections were defined by recipient or donor respiratory cultures that required treatment immediately after LTx. Primary graft dysfunction (PGD) was graded by the International Society for Heart and Lung Transplantation guidelines.^14^ Peak post-transplant lung function was defined by the highest forced expiratory value in 1 second (FEV_1_) and forced vital capacity percent predicted by 1 year. Donor:recipient height and predicted total lung capacity (pTLC) ratios were calculated for each transplant, as previously described.^15^, ^16^

For the analyses, ACs were defined as:

1. Any need for bronchoscopic balloon dilation, debridement, or stenting.
  OR
2. Any bronchoscopically diagnosed airway dehiscence.

ACs were further characterized at the time of first diagnosis according to the 2018 International Society for Heart and Lung Transplantation Consensus Statement.^11^

### Surgical and postoperative management

All donor lungs were given standard antegrade and retrograde Perfadex flush at procurement and transported at approximately 4°C. All transplants during the study period were performed on cardiopulmonary bypass. Backtable preparation was performed to trim the donor bronchi to within 2 rings of the secondary carina. All bronchial anastomoses were performed with a running 4-0 PDS along the membranous airway and interrupted, figure-of-eight 4-0 PDS for the cartilaginous portion of the airway. One gram of methylprednisolone was administered just before reperfusion of the first lung and 500 mg before the second lung. Bronchoscopy was performed before leaving the operating room to clear secretions and assess the anastomoses.

Vasopressors were titrated to a mean arterial pressure of >65 mm Hg, and fluid management decisions factored in hemodynamics, organ perfusion, and respiratory status. Right heart catheters were removed at the discretion of the attending physician. Extubation was performed as early as possible, after epidural catheter placement. Bronchoscopies were completed in all recipients before extubation and otherwise on a case-by-case basis during the initial hospitalization. Following discharge, bronchoscopies were performed on a clinically indicated basis as previously described.^17^ Standard immunosuppression and infectious prophylaxis are shown in the Supplement.

### Statistical analysis

Analysis was performed in R version 4.2.2 for Windows. Histogram and QQ plots assessed whether variables were normally distributed. Demographics, pre- and perioperative characteristics, and outcomes were summarized using the mean (standard deviation) or median (interquartile range) for numeric variables, and count (percentage) for categorical variables. Between-group differences were assessed using either *t*-test or the Wilcoxon-Mann-Whitney test for continuous variables, and the chi-square test or Fisher’s exact test for categorical variables. Kaplan-Meier survival analysis for all-cause mortality was performed using the survival package in R, with differences in survival between AC groups determined using the log-rank test. Cox proportional hazards was also performed to determine the effect of any AC on all-cause mortality, both before and after adjusting for age and diagnosis group. A *p*-value of less than 0.05 was considered statistically significant. Estimates of cumulative incidence for the competing risks of any AC and all-cause mortality without AC were estimated using the cuminc function in the tidycmprsk package, and cumulative incidence curves for each event were displayed using the ggsurvfit package.

A separate sensitivity analysis assessed for associations between pretransplant medications and ACs by different groupings as follows (Supplement):

1. Antifibrotics vs no antifibrotics.
2. Only antifibrotics, only prednisone, or multiple medications vs no medications.

## Results

A total of 145 bilateral LTx recipients were included (study flowchart shown in Figure S1). Mean age was 60.0 years and 59.3% were male (Table 1). Group A (obstructive), B (pulmonary vascular), C (suppurative), and D (restrictive) diagnoses accounted for 24 (16.6%), 13 (9.0%), 8 (5.5%), and 100 (69.0%) transplants, respectively. Idiopathic pulmonary fibrosis accounted for 43 (43.0%) of the group D diagnoses and was the most commonly transplanted individual diagnosis (specific diagnoses shown in Table S1).

**Table 1:** Demographics and Preoperative Characteristics

Characteristic	No AC (*n* = 97)	AC (*n* = 48)	Total (*n* = 145)	*p*-value
Age at lung transplant (years)	59.0 (49.0, 66.0)	61.0 (55.5, 65.0)	60.0 (51.0, 65.0)	0.4
Sex assigned at birth				0.007
Female	47 (48.5%)	12 (25.0%)	59 (40.7%)	
Male	50 (51.6%)	36 (75.0%)	86 (59.3%)	
Diagnosis group				0.057
A (Obstructive)	19 (19.6%)	5 (10.4%)	24 (16.6%)	
B (Pulmonary vascular)	9 (9.3%)	4 (8.3%)	13 (9.0%)	
C (Suppurative)	8 (8.3%)	0 (0.0%)	8 (5.5%)	
D (Restrictive)	61 (62.9%)	39 (81.2%)	100 (69.0%)	
BMI at transplant (kg/m^2^)	25.7 (22.2, 28.4)	26.4 (23.5, 28.1)	25.9 (22.6, 28.4)	0.5
CAS medical urgency	0.943 (0.340, 5.915)	0.626 (0.341, 1.806)	0.758 (0.340, 5.836)	0.5
Match LAS	40.8 (35.8, 44.2)	43.71 (37.7, 53.0)	41.0 (36.0, 48.2)	0.086
CAS/LAS tertile				0.5
First tertile	34 (35.1%)	15 (31.3%)	49 (33.8%)	
Second tertile	34 (35.1%)	14 (29.2%)	48 (33.1%)	
Third tertile	29 (29.9%)	19 (39.6%)	48 (33.1%)	
Condition at transplant				>0.9
Hospitalized, not in ICU	9 (9.3%)	4 (8.3%)	13 (9.0%)	
In ICU	7 (7.2%)	4 (8.3%)	11 (7.6%)	
Not hospitalized	81 (83.5%)	40 (83.3%)	121 (83.5%)	

Abbreviations: AC, airway complications; BMI, body mass index; CAS, composite allocation score (nearest transplant); ICU, intensive care unit; LAS, lung allocation score (at transplant).

In total, 24 recipients (16.6%) were hospitalized leading into LTx, with 11 (7.6%) in the ICU. One hundred and two (70.3%) and 43 (29.7%) were transplanted in the LAS and CAS eras, respectively, and the median match LAS and CAS medical urgency subscores were 41.0 and 0.758.

A total of 48 of 145 LTx recipients (33.1%) developed ACs, diagnosed at a mean of 77.3 days post-transplant. Fifteen (10.3%) developed anastomotic dehiscence diagnosed at a mean of 27.3 days post-transplant. Of dehiscence, 4 (26.7%) were right-sided, 4 (26.7%) left-sided, and 7 (46.7%) were bilateral, with all involving <25% of the airway circumference. Of recipients with airway dehiscence, associated complications included empyema in 8 (53.3%), bacteremia in 3 (20%), pneumothorax in 9 (60%), and bronchopleural fistula in 4 (26.7%). There were no cases of fistulization to the vasculature or other structures. Six of 15 (40.0%) recipients with anastomotic dehiscence went on to develop airway stenosis, and 10 of 15 (66.7%) died during their initial hospitalization.

A total of 39 LTx recipients developed airway stenoses, of which 12 (30.8%) were right-sided, 9 (23.1%) left-sided, and 18 (46.2%) were bilateral; 20 (51.2%) stenoses involved only the anastomotic site(s), 3 (7.7%) only the lobar/segmental bronchi, and 16 (41.0%) the anastomotic plus lobar/segmental bronchi.

### Pretransplant risk factors

LTx recipients with ACs were more often male than those without (75.0% vs 51.6%, respectively, *p* = 0.007). There were no significant differences in age, pretransplant hospitalization status, or LAS/CAS (by value or tertile) (Table 1). Thirty-nine of 48 (81.3%) ACs occurred in patients transplanted for group D diagnoses, but diagnosis was not statistically significantly associated with AC development (*p* = 0.057).

A total of 71 (49.0%) patients were on corticosteroids leading into LTx, of whom 66 (93.0%) were on a prednisone equivalent ≤20 mg daily. Among patients on pretransplant prednisone, daily and weight-based doses were not significantly associated with the development of ACs (*p* = 0.4 and >0.9, respectively; Table 2).

**Table 2:** Pretransplant Medications

Medications	No AC (*n* = 97)	AC (*n* = 48)	Total (*n* = 145)	*p*-value
Grouping^a^				0.7
Prednisone alone	11 (11.3%)	6 (12.5%)	17 (11.7%)	
Other IS alone	5 (5.2%)	3 (6.3%)	8 (5.5%)	
Antifibrotic alone	15 (15.5%)	5 (10.4%)	20 (13.8%)	
Antifibrotic + prednisone	11 (11.3%)	8 (16.7%)	19 (13.1%)	
Antifibrotic + other IS	0 (0.0%)	1 (2.1%)	1 (0.7%)	
Prednisone + other IS	14 (14.4%)	7 (14.6%)	21 (14.5%)	
Prednisone + other IS + antifibrotic	8 (8.3%)	6 (12.5%)	14 (9.7%)	
None	33 (34.0%)	12 (25.0%)	45 (31.0%)	
Prednisone dose in full cohort (mg)				0.3
0	53 (54.6%)	21 (43.8%)	74 (51.0%)	
5	8 (8.3%)	2 (4.2%)	10 (6.9%)	
7.5	1 (1.0%)	0 (0.0%)	1 (0.7%)	
10	15 (15.5%)	13 (27.1%)	28 (19.3%)	
15	6 (6.2%)	1 (2.1%)	7 (4.8%)	
17	1 (1.0%)	0 (0.0%)	1 (0.7%)	
17.5	1 (1.0%)	0 (0.0%)	1 (0.7%)	
20	9 (9.3%)	9 (18.8%)	18 (12.4%)	
30	1 (1.0%)	0 (0.0%)	1 (0.7%)	
40	1 (1.0%)	0 (0.0%)	1 (0.7%)	
55	0 (0.0%)	1 (2.1%)	1 (0.7%)	
60	1 (1.0%)	0 (0.0%)	1 (0.7%)	
1250	0 (0.0%)	1 (2.1%)	1 (0.7%)	
Median daily prednisone dose (mg/day)^b^	10.0 (10.0, 20.0)	10.0 (10.0, 20.0)	10.0 (10.0, 20.0)	0.4
Median weight-based prednisone dose (mg/kg)^b^	0.16 (0.12, 0.25)	0.15 (0.12, 0.26)	0.16 (0.12, 0.25)	>0.9

Abbreviations: AC, airway complications; IS, immunosuppressant.

a Pretransplant medications in mutually exclusive groups.

b Median daily and weight-based prednisone doses are among the 71 lung transplant recipients on prednisone.

Forty-four recipients (30.3%) were on other immunosuppressants leading into LTx, of whom 34 (77.3%) were on mycophenolate. Nonmycophenolate immunosuppressants included tacrolimus (*n* = 4), methotrexate (*n* = 2), azathioprine (*n* = 2), and others (*n* = 8). A total of 54 patients (54.0% of group D diagnoses) were on pretransplant antifibrotics. There were no significant associations between pretransplant prednisone, other immunosuppressants, or antifibrotics, either alone or in any combination, and development of ACs (*p* = 0.7) (Tables 2 and S2).

### Transplant-associated risk factors

There were no significant associations between donor demographics and ACs (Table 3). Recipients with ACs did have statistically significantly lower mean donor:recipient height (1.00 vs 1.01; *p* = 0.024) and pTLC (0.97 vs 1.02; *p* = 0.007) ratios compared to those without ACs, though these differences were very modest.

**Table 3:** Perioperative and Postoperative Characteristics

Characteristic	No AC (*n* = 97)	AC (*n* = 48)	Total (*n* = 145)	*p*-value
Donor age (years)	31.0 (24.0, 38.0)	33.0 (24.8, 43.0)	32.0 (24.0, 39.0)	0.4
Donor sex assigned at birth				0.7
Female	48 (49.5%)	22 (45.8%)	70 (48.3%)	
Male	49 (50.5%)	26 (54.2%)	75 (51.7%)	
Donor ≥ 20 pack-year smoking	6 (6.2%)	4 (8.3%)	10 (6.9%)	0.7
Donor/recipient sex mismatch	33 (34.0%)	18 (37.5%)	51 (35.2%)	0.7
Donor/recipient height ratio	1.01 (0.04)	1.00 (0.04)	1.01 (0.04)	0.024
Donor/recipient pTLC ratio	1.02 (0.92, 1.11)	0.97 (0.85, 1.04)	1.00 (0.91, 1.09)	0.007
Total ischemic time (hours)	4.9 (4.0, 6.0)	5.0 (4.3, 5.6)	4.9 (4.1, 5.6)	>0.9
CPB duration (min)	179.0 (163.0, 209.0)	187.5 (171.8, 210.0)	183.0 (166.0, 209.0)	0.3
Transfusions—OR (units)	6.0 (3.0, 11.0)	6.0 (2.8, 11.3)	6.00 (3.0, 11.0)	0.9
Transfusions—first 24 hours (units)	0.0 (0.0, 1.0)	0.0 (0.0, 1.0)	0.00 (0.0, 1.0)	0.4
Postoperative ECMO	3 (3.1%)	4 (8.3%)	7 (4.8%)	0.2
Bacterial infection^a^	76 (78.4%)	38 (79.2%)	114 (78.6%)	>0.9
Fungal infection^a^	58 (59.8%)	23 (47.9%)	81 (55.9%)	0.2
PGD grade at 72 hours				0.5
0	22 (22.7%)	13 (27.1%)	35 (24.1%)	
1	52 (53.6%)	20 (41.7%)	72 (49.7%)	
2	10 (10.3%)	5 (10.4%)	15 (10.3%)	
3	13 (13.4%)	10 (20.8%)	23 (15.9%)	
PEEP (cmH_2_O)^b^				0.14
5	60 (61.9%)	31 (64.6%)	91 (62.8%)	
6	2 (2.1%)	0 (0.0%)	2 (1.4%)	
8	13 (13.4%)	10 (20.8%)	23 (15.9%)	
10	20 (20.6%)	4 (8.3%)	24 (16.6%)	
11	0 (0.0%)	1 (2.0%)	1 (0.7%)	
12	2 (2.1%)	2 (4.1%)	4 (2.8%)	
PIP (cmH_2_O)^b^	27.6 (4.3)	28.4 (4.8)	27.9 (4.5)	0.3
Pplat (cmH_2_O)^b^	22.5 (4.2)	22.8 (3.8)	22.6 (4.1)	0.7
Length of mechanical ventilation (days)	2.0 (1.0, 5.0)	3.0 (2.0, 11.3)	2.0 (1.0, 6.0)	0.043
Required tracheostomy^c^	10 (10.3%)	9 (18.8%)	19 (13.1%)	0.2
Initial hospitalization LOS (days)	23.0 (16.0, 36.0)	27.0 (16.8, 45.8)	23.0 (16.0, 39.0)	0.2
VIS—arrival	6.0 (3.0, 10.1)	6.5 (2.8, 13.8)	6.0 (3.0, 10.9)	0.3
VIS—24 max	13.0 (8.0, 19.0)	18.0 (9.8, 22.2)	14.2 (8.0, 20.2)	0.021
Max postoperative lactate	6.8 (4.8, 10.2)	9.1 (6.8, 11.0)	7.6 (5.3, 10.7)	0.017
Required HD	10 (10.3%)	11 (22.9%)	21 (14.5%)	0.042
Net weight gain				
OR (kg)	7.3 (4.4, 11.0)	6.8 (4.9, 9.0)	7.2 (4.4, 10.5)	0.3
OR (% body weight)	9.9 (6.3, 15.9)	10.4 (5.0, 12.4)	10.3 (6.1, 14.6)	0.3
At 24 hours (kg)	8.7 (6.2, 12.5)	10.3 (7.8, 12.9)	9.2 (6.6, 12.8)	0.072
At 24 hours (% body weight)	12.7 (8.0, 18.8)	14.2 (10.5, 17.4)	13.0 (8.8, 17.8)	0.3
At 48 hours (kg)	9.2 (5.8, 12.2)	8.6 (6.8, 12.8)	9.0 (6.0, 12.20)	0.6
At 48 hours (% body weight)	11.8 (7.8, 17.7)	12.6 (8.3, 17.2)	12.0 (8.1, 17.5)	0.9
At 72 hours (kg)	8.1 (4.6, 10.9)	7.8 (4.6, 12.1)	8.0 (4.6, 11.3)	>0.9
At 72 hours (% body weight)	10.9 (6.6, 16.0)	10.2 (5.9, 16.0)	10.7 (6.5, 16.0)	0.6
Net intake—output				
First 24 hours (liters)	7.9 (6.0, 10.8)	9.6 (6.9, 12.5)	8.3 (6.3, 11.6)	0.2
First 48 hours (liters)	8.9 (6.6, 11.6)	10.6 (8.7, 15.1)	9.5 (6.8, 13.1)	0.028
First 72 hours (liters)	8.0 (4.9, 11.3)	8.8 (5.8, 14.1)	8.0 (5.1, 11.9)	0.2
Missing values	11	3	14	

Abbreviations: AC, airway complications; CPB, cardiopulmonary bypass; ECMO, extracorporeal membrane oxygenation; HD, hemodialysis (during initial hospitalization); LOS, length of stay; OR, operating room; PEEP, positive end-expiratory pressure; PIP, peak inspiratory pressure; Pplat, plateau pressure; PGD, primary graft dysfunction; pTLC, predicted total lung capacity; VIS, vasopressor-inotropic score

a Infections were defined by culture positivity and receipt of treatment immediately after transplant.

b PEEP, PIP, and Pplat values represent the mean of highest pressures in the first 24 hours.

c Indicated requirement for tracheostomy during initial hospitalization.

There were no significant differences in ischemic times, cardiopulmonary bypass times, intra-/postoperative blood transfusion requirements, or need for postoperative extracorporeal membrane oxygenation between LTx recipients who did vs did not develop ACs (Table 3). A total of 114 (78.6%) and 81 (55.9%) recipients were treated peri-/postoperatively for bacterial and fungal infections, respectively, without differences in the development of ACs (specific infections shown in Table S3).

From a respiratory standpoint, 10 (20.8%) LTx recipients with ACs had PGD grade 3 at 72 hours compared to 13 (13.4%) of those without ACs, but there were no significant differences in PGD grade (*p* = 0.5). Similarly, there were no differences in mechanical ventilation parameters in the first 24 hours, including highest peak pressures, plateau pressures, or amount of positive end-expiratory pressure (PEEP) required, though recipients with ACs did have longer mean time on the ventilator (3.0 vs 2.0 days, respectively, *p* = 0.043).

Importantly, recipients who developed ACs were more likely to have had early postoperative hypotension and hypoperfusion compared to those who did not, as shown by significantly higher peak VIS (18.0 vs 13.0, respectively, *p* = 0.021), lactate levels (9.1 vs 6.8, *p* = 0.017), and need for hemodialysis during their transplant hospitalization (22.9% vs 10.3%, *p* = 0.042) (Table 3).

**Figure 1:**
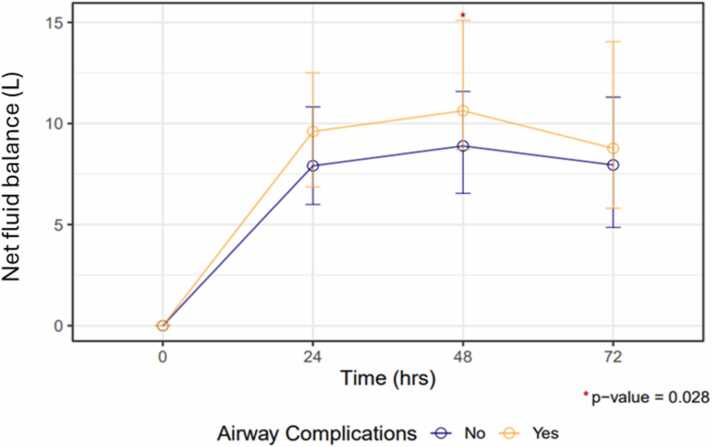
Net fluid balance (liters) by time post lung transplant and airway complication status.

Fluid resuscitation was assessed by body weight and net fluid balance over the first 72 postoperative hours. Weight increased similarly in recipients with and without ACs (7.8 vs 8.1 kg increase at 72 hours, respectively, *p* > 0.9). Recipients who developed ACs received a higher net positive fluid balance at 24, 48, and 72 hours post-transplant (Figure 1); this difference was statistically significant at the 48 hours timepoint, at which time recipients with ACs were net positive 10.6 liters, compared to 8.9 liters positive in those without ACs (*p* = 0.028).

### Outcomes

Airway stenoses were treated mainly with serial balloon dilations. Two recipients received airway stents. Recipients with dehiscence in all cases received systemic antimold therapy and culture-directed antibacterials, as well as tube thoracostomy for associated empyemas, pneumothoraces, or bronchopleural fistula. One dehiscence over the study period that resulted in bronchopleural fistula underwent pleural patch repair using a latissimus muscle flap. In total, recipients with ACs underwent a mean of 5.6 bronchoscopic interventions over the first post-transplant year.

**Figure 2:**
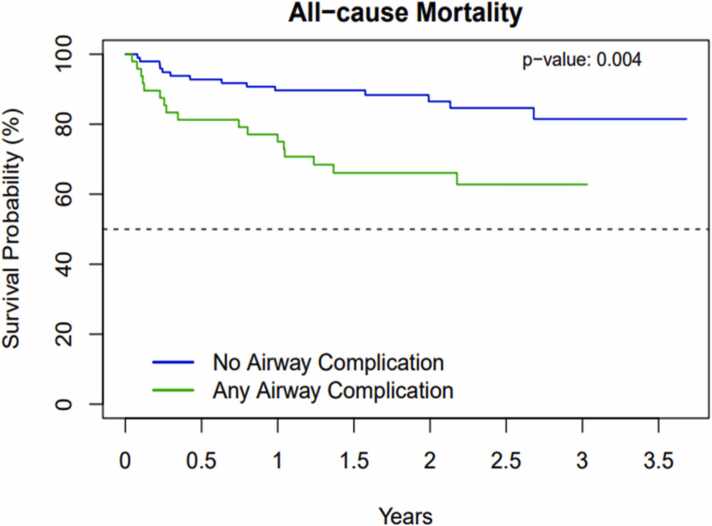
Kaplan-Meier survival curves demonstrating post lung transplant survival by airway complication status.

**Figure 3:**
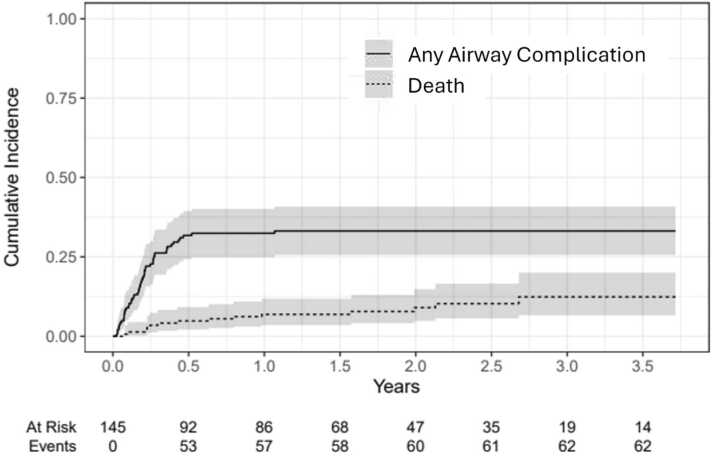
Cumulative incidence for the competing risks of any airway complication and death without airway complication.

Recipients with ACs had significantly higher mortality (Figure 2). Survival at 6 months, 1 year, and 3 years among those with ACs was 81.2%, 75.0%, and 62.8%, respectively, compared to 92.8%, 89.7%, and 81.5% among those without. In the univariate Cox proportional hazards model, recipients with ACs had a hazard ratio of death of 2.74 (95% CI [1.35, 5.55], *p* = 0.004) compared to those without ACs. In the multivariate Cox proportional hazards model (including age and diagnostic group), recipients with ACs had a hazard ratio of death of 3.39 (95% CI [1.54, 7.43], *p* = 0.002) compared to those without ACs, holding age and diagnostic group constant. Figure 3 and Table S4 demonstrate the cumulative incidence by time post-transplant for the competing risks of any AC and all-cause mortality without AC.

Among 130 recipients with pulmonary function testing, those with ACs had lower peak FEV_1_% predicted than those without (74.8% vs 86.3%, respectively, *p* = 0.013). Peak forced vital capacity percent was also lower among those with ACs, but the difference was not statistically significant (77.3% vs 85.5%, respectively, *p* = 0.14).

## Discussion

The goal of this study was to analyze modifiable risk factors for ACs after LTx in a modern-day cohort. We demonstrated significant associations between early post lung transplant hypotension, hypoperfusion, and the subsequent development of ACs. This study also evaluated the composite risks of pretransplant lung disease–modifying medications, and we found that pretransplant corticosteroids (including by dose), other immunosuppressants, or antifibrotics, either alone or in any combination, were not associated with AC development. ACs were associated with worsened clinical outcomes after LTx, including increased mortality.

LTx outcomes have gradually improved, but many surgical, immunologic, and medical complexities contribute to overall survival that lags behind other solid organs.^18^, ^19^, ^20^ ACs are among the most important surgical complications, occurring in up to 35% of recipients and carrying a great deal of morbidity.^1^, ^2^ The impact of ACs on survival has varied in prior studies, but our results certainly support a strong association with worse outcomes, including a need for recurrent interventions, lower survival at all time points, and the added finding of lower peak FEV_1_ indicating baseline lung allograft dysfunction.

An important question is whether ACs develop simply as a product of surgical factors combined with the degree of illness for a given recipient, or whether any nonsurgical perioperative factors can be optimized to reduce risk. Understanding AC pathogenesis is crucial in answering this question, which is felt to arise from surgical disruption of the bronchial artery circulation, leaving airway perfusion dependent on retrograde filling from the low-pressure, deoxygenated pulmonary circulation via the submucosal plexus.^11^, ^21^ It is possible that postoperative events could play an important role by further reducing airway perfusion,^11^ but data describing these events are quite limited. One study from 1990 showed that 5 of 8 LTx recipients with severe postoperative hypotension developed ACs,^22^ but surgical techniques have evolved significantly since that time. One study from 1991 used a canine LTx model to demonstrate reduced airway mucosal blood flow resulting from higher mechanical ventilation pressures/PEEP,^23^ but to our knowledge, there are no human studies describing this phenomenon.

Our results support the notion of reduced vascular flow predisposing to ACs in LTx recipients who may be otherwise susceptible. Similar to other studies, we found that men were more likely to develop ACs, as well as a trend toward increased risk for group D (restrictive) diagnoses. It could be that these subgroups are more prone, and in the face of early hypoperfusion, as marked in our study by higher postoperative vasopressor requirements, lactate levels, and degree of ischemic kidney injury, airway ischemia, and impaired healing may result. A natural question is whether the risk of hypoperfusion could be mitigated by more liberal fluid resuscitation in the early postoperative period. We initially hypothesized that LTx recipients who develop ACs may have been more often “under-resuscitated” during and/or after transplant. Our results, however, show that recipients who developed ACs had a significantly positive early fluid balance that was at least as high as those who did not, suggesting instead that this cohort may simply be more inflamed and/or vasoplegic postoperatively. It is challenging to know how best to manage this common problem, but it should be noted that, during the study period, all lung transplants at our center were supported intraoperatively with cardiopulmonary bypass. Our intraoperative support should be noted when interpreting our results, and further studies are needed to better understand contributors to vasoplegia after LTx, including whether the choice of bypass vs other modalities of support may impact the degree of postoperative airway hypoperfusion.

For candidates approaching LTx, our analysis focused on lung disease–modifying medications for a few reasons. First, it is conceivable that corticosteroids, other immunosuppressants, and/or antifibrotics may impact airway healing either directly or by predisposing to early airway infection, and we hypothesized that pretransplant medications could represent modifiable risk. Second, while a few studies have addressed the risk of individual medication classes before LTx, there remain many unknowns. For example, corticosteroids were found in older studies to increase the risk for post-transplant ACs,^24^ but the impact of specific dosing regimens in the modern era remains uncertain.^10^, ^25^, ^26^ Literature in the early 2000s suggested that mammalian target of rapamycin inhibitors increased the risk of anastomotic dehiscence,^27^, ^28^ but to our knowledge, there are no studies documenting AC risk with other classes of pretransplant immunosuppressants. Lastly, while recent studies have not shown associations between antifibrotics and ACs,^29^, ^30^, ^31^, ^32^ to our knowledge, there is no literature documenting the risks when used in combination with corticosteroids or other immunosuppressants, an approach that is more frequently being used in the modern era as these drugs have been approved for broader indications. Our highly granular dataset included many patients on pretransplant disease–modifying medications, and we did not find any associations with ACs even among patients on triple-combination therapy. While our findings are reassuring, further studies will be needed to evaluate whether AC risk can be mitigated using other strategies before LTx.

Our study has several limitations. First, although our dataset was highly detailed, this was a single-center study with relatively small numbers. This was arguably most important in our analysis of pretransplant medications and post-transplant mechanical ventilation parameters, where the total numbers in each subgrouping were relatively low and most patients were on low doses of prednisone, therefore reducing our power to detect small differences. Sensitivity analyses pooling patients into larger medication exposure groups (“any anti-fibrotic” vs none) were also negative, but our findings should be interpreted within the context of these limitations. The relatively small number of events (deaths and ACs) also limited our ability to assess for potential confounding, although our adjusted analysis also showed a significant association between ACs and mortality.

Second, in an attempt to capture a contemporary cohort that received a modern paradigm of pretransplant lung disease treatments, our study bridged the transition from the LAS to CAS. Pretransplant allocation score is often used as a surrogate for disease severity, and we did not find any differences in recipients who developed ACs, but the challenges in comparing 2 different allocation scoring systems should be appreciated. We were able to use a novel approach comparing LAS and CAS tertiles that confirmed our findings, and this method may serve as a model for future studies that bridge the transition between allocation score eras. Third, in regard to fluid resuscitation, although net fluid balance was not lower among those with ACs, it is important to recognize that this finding cannot fully rule out the possibility that additional fluids could have lessened risks; while it seems likely that those with ACs were simply more vasoplegic and thus no longer fluid responsive, optimal post-transplant resuscitation strategies cannot be fully delineated in this retrospective analysis and further studies are needed. Finally, it should be noted that we used a fairly inclusive definition and therefore the percentage of patients with ACs was on the higher end of previously reported ranges. Arguably, this would bias toward the null hypotheses and thus our conclusions remain supported, but the definition and percentage of recipients with ACs should be considered when interpreting our results.

Despite these limitations, our robust dataset allowed us to demonstrate that early postoperative hypotension with resulting hypoperfusion is a significant risk factor for ACs after LTx. ACs significantly increase morbidity and mortality, and further studies are needed to better delineate strategies to mitigate risk.

## CRediT authorship contribution statement

All listed authors contributed to the design of the work or data acquisition, analysis, or interpretation. S.G.K., K.J.R., and R.F. drafted the article. All other authors contributed to reviewing the work for important intellectual content and final approval. All authors agree to be accountable for all aspects of the work.

## Disclosure statement

The authors declare that they have no known competing financial interests or personal relationships that could have appeared to influence the work reported in this paper.

Kathleen J. Ramos: 10.13039/100000002NIH P30 DK089507. Erika D. Lease: LEASE17AB3, NIH/10.13039/100000050NHLBI R33HL158811.

## Footnotes

Appendix A Supplementary data associated with this article can be found in the online version at doi:10.1016/j.jhlto.2025.100315.
