# Supplementary material for: Airway complications after lung transplantation: Perioperative risk factors and clinical outcomes
Source: JHLT Open. 2025 Jun 6;9:100315. doi: 10.1016/j.jhlto.2025.100315 (PMC12240133; doi:10.1016/j.jhlto.2025.100315)
Supplement: Supplementary file 1 — Supplementary material [file mmc1.docx]

**Airway Complications After Lung Transplantation: Peri-operative Risk Factors and Clinical Outcomes**

**ONLINE DATA SUPPLEMENT**

**Authors:**

Siddhartha G. Kapnadak, MD^1^, Kathleen J. Ramos, MD, MS^1^, Rachel Flodin, MS^2^, Sanaa Mansoor, MD^2^, Kyle Bilodeau, MD^2^, Peter Beidler, BS^2^, Erika D. Lease, MD^1^, Ryan Thomas, BS^2^, Richard Dubois, MD^2^, Jay Pal, MD, PhD^2^, Michael S. Mulligan, MD^2^

^1^Division of Pulmonary, Critical Care, and Sleep Medicine, Department of Medicine

^2^Division of Cardiothoracic Surgery, Department of Surgery

University of Washington

**Standard Immunosuppression and infectious prophylaxis:**

During the entirety of the study period the standard immunosuppression included basiliximab (post-operative days 0 and 4), mycophenolate mofetil 1000mg twice daily, methylprednisolone 125mg/kg every 12 hours for six doses followed by prednisone (starting at 0.7mg/kg/daily, then weaning by 0.1mg/kg/daily every two weeks until 10mg daily, then weaning to 5mg daily at nine months post-transplant). Tacrolimus was initiated in the first 72 hours of transplant targeting an initial goal between 8-12 ng/mL.

Initial routine infectious prophylaxis included cefazolin, trimethoprim/sulfamethoxazole, ganciclovir (or acyclovir if cytomegalovirus donor seronegative, recipient seronegative), oral nystatin, and inhaled amphotericin. Additional antibacterial or antifungal coverage was directed towards any positive pre-/day-of-transplant respiratory cultures in the recipient or donor.

**Supplementary Results:**

Figure 1: Study Flowchart

**
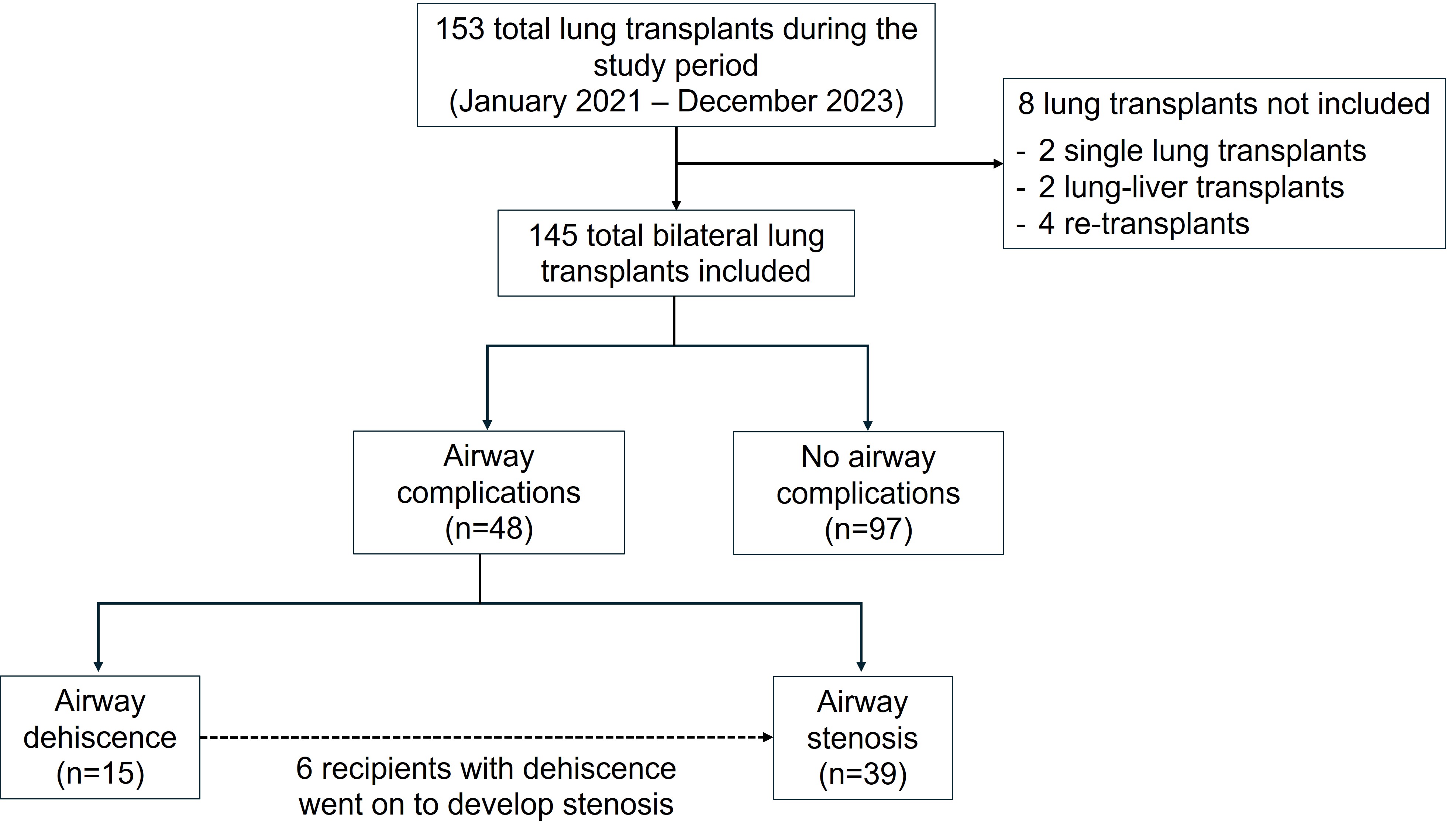
**

| **Table S1: Specific pre-lung transplant diagnoses by diagnosis group** | | |
| --- | --- | --- |
| Diagnosis group | Specific diagnosis | Number and % of diagnosis group |
| A (n=24) | Chronic obstructive pulmonary disease | 15 (62.5%) |
|  | Lymphangioleiomyomatosis | 5 (20.8%) |
|  | Alpha-1 antitrypsin | 2 (8.3%) |
|  | Other | 2 (8.3%) |
| B (n=13) | Pulmonary arterial hypertension | 13 (100.0%) |
| C (n=8) | Cystic fibrosis | 8 (100.0%) |
| D (n=100) | Idiopathic pulmonary fibrosis  Hypersensitivity pneumonitis  COVID-19  Non-specific interstitial pneumonitis  Scleroderma  Polymyositis  Rheumatoid arthritis  Sarcoidosis  Other | 43 (43.0%)  10 (10.0%)  6 (6.0%)  4 (4.0%)  4 (4.0%)  3 (3.0%)  3 (3.0%)  3 (3.0%)  24 (24.0%) |

A separate sensitivity analysis assessed for associations between pre-transplant medications and ACs by different groupings:

1. Antifibrotics versus no antifibrotics.
2. Only antifibrotics, only prednisone, or multiple medications versus no medications.

Univariate logistic regression was performed using Firth's bias-reduced logistic regression using the logistf package in R, with the penalized profile log-likelihood method used to calculate the confidence intervals and the p-values. Results:

| **Table S2: Univariate Firth’s bias-reduced logistic regression analysis of association of medication groupings with the outcome of any airway complication** | | | |
| --- | --- | --- | --- |
|  | **Odds Ratio** | **95% CI** | **p-value** |
| MODEL 1 (Reference group: No antifibrotics) |  |  |  |
| (Intercept) | 0.7091 | (0.3915, 1.2598) |  |
| Antifibrotics - yes | 0.8380 | (0.3761, 1.8636) | 0.664 |
|  |  |  |  |
| MODEL 2 (Reference group: No medications) |  |  |  |
| (Intercept) | 1.2222 | (0.3468, 4.5455) |  |
| Antifibrotic only | 0.2903 | (0.0557, 1.3844) | 0.121 |
| Prednisone only | 0.6000 | (0.1079, 3.1522) | 0.545 |
| On multiple agents/Other | 0.5647 | (0.1385, 2.1925) | 0.405 |

| **Table S3: Specific infections requiring treatment after lung transplantation** | | |
| --- | --- | --- |
| Group | Specific organisms | Number of recipients |
| Bacterial  (n=114 total recipients) | MSSA | 61 |
|  | *Klebsiella* sp. | 16 |
|  | *Pseudomon*as aeruginosa | 16 |
|  | MRSA | 15 |
|  | *Enterobacter* sp. | 10 |
|  | *Stenotrophomonas maltophilia* | 10 |
|  | *Serratia* sp. | 7 |
|  | *Streptococcus pneumoniae* | 7 |
|  | Haemophilus *influenzae* | 5 |
|  | Acinetobact*er* sp. | 5 |
|  | *Enterococcus sp.* | 4 |
|  | *Escherichia coli* | 4 |
|  | *Burkholderia multivorans* | 3 |
|  | *Citrobacter* sp. | 3 |
|  | *Achromobacter* sp. | 2 |
|  | *Corynebacterium* sp. | 2 |
|  | Other | 8 |
| Non-tuberculous mycobacteria (n=1 total recipient) | Mycobacterium avium complex | 1 |
| Fungal  (n=81 total recipients) | *Candida* sp. | 78 |
|  | *Aspergillus* sp. | 10 |
|  | *Scedosporium apiospermum* | 2 |
|  | Other | 4 |

| **Table S4: Cumulative incidence estimates for the competing risks of any airway complication and death** | | |
| --- | --- | --- |
|  | **Outcome** | |
| **Time post-transplant** | **Airway Complication** | **Death** |
| 3 months | 22.76% (16.30%, 29.88%) | 3.45% (1.29%, 7.39%) |
| 6 months | 31.72% (24.30%, 39.39%) | 4.83% (2.13%, 9.21%) |
| 1 year | 32.41% (24.93%, 40.11%) | 6.90% (3.52%, 11.83%) |
| 1.5 years | 33.14% (25.58%, 40.86%) | 6.90% (3.52%, 11.83%) |
| 2 years | 33.14% (25.58%, 40.86%) | 9.03% (4.83%, 14.84%) |
| 2.5 years | 33.14% (25.58%, 40.86%) | 10.29% (5.61%, 16.61%) |
| 3 years | 33.14% (25.58%, 40.86%) | 12.38% (6.62%, 20.05%) |
| 3.5 years | 33.14% (25.58%, 40.86%) | 12.38% (6.62%, 20.05%) |
